# Small intestinal permeability in older adults

**DOI:** 10.14814/phy2.281

**Published:** 2014-04-23

**Authors:** Luzia Valentini, Sara Ramminger, Verena Haas, Elisa Postrach, Martina Werich, André Fischer, Michael Koller, Alexander Swidsinski, Stefan Bereswill, Herbert Lochs, Jörg‐Dieter Schulzke

**Affiliations:** 1 Department of Gastroenterology and Hepatology, Section of Nutritional Medicine, Charité – Universitätsmedizin Berlin, Berlin, Germany; 2 Department of Microbiology and Hygiene, Charité – Universitätsmedizin Berlin, Berlin, Germany; 3 Center for Clinical Studies, University Hospital Regensburg, Regensburg, Germany; 4 Medical University Innsbruck, RectorateInnsbruck, Austria

**Keywords:** Aging, cardiovascular risk, gut leakiness, small intestine, sugar test

## Abstract

It is not yet clear whether intestinal mucosal permeability changes with advancing age in humans. This question is of high importance for drug and nutrition approaches for older adults. Our main objective was to answer the question if small intestinal barrier integrity deteriorates with healthy aging. We conducted a cross‐sectional study including the pooled data of 215 nonsmoking healthy adults (93 female/122 male), 84 of whom were aged between 60 and 82 years. After a 12‐h fast, all participants ingested 10 g of lactulose and 5 g of mannitol. Urine was collected for 5 h afterwards and analyzed for test sugars. The permeability index (PI = lactulose/mannitol) was used to assess small intestinal permeability. Low‐grade inflammation defined by high‐sensitivity C‐reactive protein ≥1 mL/L and kidney function (estimated glomerular filtration rate) were determined in the older age group. The PI was similar in older compared to younger adults (*P *=**0.887). However, the urinary recovery of lactulose and mannitol was lower in the older adults and this change was neither associated with urinary volume nor glomerular filtration rate. The PI was not significantly correlated with low‐grade inflammation or presence of noninsulin‐dependent type 2 diabetes. However, it significantly deteriorated in the copresence of both conditions compared to low‐grade inflammation alone (*P *=**0.043) or type 2 diabetes alone (*P *=**0.015). Small intestinal mucosal barrier does not deteriorate with age per se. But low‐grade inflammation coupled with minor disease challenges, such as type 2 diabetes, can compromise the small intestinal barrier.

## Abstract (precis)

Until now, it has not been clear if the small intestinal mucosal barrier deteriorates with age per se. We investigated the pooled data of 215 nonsmoking healthy adults, 84 of whom were aged between 60 and 82 years and found similar intestinal permeability results in all age classes. However, in participants with low‐grade inflammation coupled with type 2 diabetes the small intestinal integrity was compromised.

## Introduction

Intestinal permeability indicates the mucosal barrier integrity and describes the paracellular leakiness of the intestinal lining. An intact intestinal barrier prevents the permeation of antigens, endotoxins, pathogens, and other proinflammatory substances into the human body, whereas intestinal dysintegrity can trigger systemic inflammation and disease (Soeters et al. 2007; Britton and McLaughlin 2013; Suzuki 2013). It is clearly evidenced that intestinal permeability is increased in a number of diseases, such as inflammatory bowel disease, celiac disease, food intolerance, allergy, malnutrition, and rheumatoid arthritis (Keita and Soderholm 2010).

Despite knowledge in disease, it is still unclear whether the normal, physiological process of aging itself worsens the integrity of the intestinal mucosa (Meier and Sturm 2009; Britton and McLaughlin 2013). With steadily increasing numbers of older people worldwide, answering this question becomes more important.

First, it is relevant in the context of gastrointestinal absorption of medical drugs (Meier and Sturm 2009). Second, a higher intestinal permeability in older age might contribute to explain the phenomenon of inflamm‐aging. Inflamm‐aging is the chronic low‐grade inflammation typical of aging which is described as complex interactions of NFkB pathways (Franceschi et al. 2007; Cevenini et al. 2013). Third, an increased intestinal permeability with age can also be important in the pathoetiology of cardiovascular diseases, because the risk to suffer a myocardial infarct or stroke increases in the presence of a low‐grade inflammation (Kalogeropoulos et al. 2012). Low‐grade inflammation associated to cardiovascular risk is mechanistically different from inflamm‐aging but can potentially also be triggered by increased leakiness of the gut.

Low‐grade inflammation is mostly defined by serum concentrations of high‐sensitivity C‐reactive protein (hsCRP; Kalogeropoulos et al. 2012). HsCRP is conventional C‐reactive protein (CRP) in low concentrations up to about 10 mg/L. The upper reference threshold for hsCRP is 1 mg/L (Pearson et al. 2003). The very mild inflammation identified by hsCRP is caused by obesity or atherosclerotic lesions and not by infection or conventional disease‐associated inflammation. Even such an insignificant increase identified by hsCRP concentration carries a twofold higher risk of myocardial infarction or stroke (Pearson et al. 2003). About 50% of older people are affected by low‐grade inflammation operationalized by hsCRP (Imhof et al. 2003; Ahmadi‐Abhari et al. 2013). Knowing more about the characteristics of permeability, changes in advanced age may help decide if the intestinal barrier should become a prime target for reducing low‐grade inflammation.

So far, only two small investigations have evaluated the intestinal permeability in healthy older people (Beaumont et al. 1987; Saltzman et al. 1995). These two investigations included 26 volunteers, but this number is too low to draw firm conclusions. A third study (Saweirs et al. 1985) included 32 older hospital patients who were not further defined, rendering the result inconclusive for aging per se.

Consequently, the data currently available are inadequate to reliably answer the question if intestinal barrier deteriorates with advancing age. To find an answer to this question was the primary aim of our study. We additionally investigated if intestinal permeability is associated with low‐grade inflammation common in older age.

## Methods

### Participants

The total sample consisted of 215 free living, healthy, and nonsmoking Caucasian volunteers (93 women/122 men). Of those, data from 134 adults were derived from previous investigations conducted by the authors in the same center between 2005 and 2008 (Valentini et al. 2008, 2009; Haas et al. 2009, 2009). Only four adults of this historic group were aged 60 years and more. Therefore, we additionally recruited 81 older adults prospectively in the year 2010 (registered at clinicaltrials.gov as NCT01218165).

All participants gave written informed consent, and the ethics committee of the Charite Universitätsmedizin Berlin approved each study. The approvals of the three historic studies from which the younger age group was pooled included the use of results for the present analysis. All studies were conducted according to the Declaration of Helsinki of 1975, as revised in 2008.

The health history of every participant was obtained by an interview, a questionnaire, and a physical examination. All participants were nonsmoking and well nourished according to standard anthropometric criteria. All participants followed the same instructions for the preparation and implementation of the permeability tests.

#### The younger age group (*n *=**130, 34 ± 11 years, range 19–59 years)

The 130 participants (89 women/41 men) of the younger age group were pooled from the original reference population for permeability values of the Department of Gastroenterology and Hepatology at the Charité Universitätsmedizin Berlin (Haas et al. 2009) and the control populations of two previous studies conducted at our center (Valentini et al. 2008, 2009).

#### The older age group (*n *=**85, 68 ± 4 years, range 60–82 years)

The older age group consisted of 81 healthy and free living men, who were prospectively recruited for an intervention study on healthy aging that was registered at clinicaltrials.gov as NCT01218165. All men were screened for normal routine blood parameters (hematologic parameters), ASAT (aspartate‐aminotransferase), ALAT (alanine‐aminotransferase), total bilirubin, direct bilirubin, creatinine, sodium, and potassium. Intake of statins or antihypertensive medication was allowed, whereas the acute or chronic intake of antibiotics, anti‐inflammatory, or analgesic medication was not permitted.

Additionally, four women of the permeability reference population of the Charité Universitätsmedizin Berlin (Haas et al. 2009) aged >60 years were allocated to the older age group for comparison with the younger age group.

Health in older age was more broadly defined and included also older people with noninsulin‐dependent type 2 diabetes, drug‐treated hypertension, or drug‐treated hypercholesteremia. Patients with type 2 diabetes have been shown to have normal intestinal permeability (Secondulfo et al. 1999); thus, no bias was expected by integrating type 2 diabetics. Exclusion criteria were all other relevant chronic or acute organ, hematologic, inflammatory, or metabolic diseases.

### Permeability tests

All tests were conducted at the permeability laboratory of the Department of Gastroenterology and Hepatology at the Charité‐Universitätsmedizin Berlin. All reported tests were analyzed by the same person (MW).

The intestinal integrity can be noninvasively investigated by oral intake and subsequently by urinary recovery of nonmetabolizable test sugars (Bjarnason et al. 1995). This simple test principle requires one monosaccharide and one disaccharide. The monosaccharide with an average molecular mass of 182 daltons is, in theory, small enough to pass through intact intestinal epithelial tight junctions. Thus, the monosaccharide reflects the total area of exposed tight junctions independent of any epithelial barrier dysfunction (Amasheh et al. 2005). It is the reference for the absorption area or the amount of villous tight junctions present in the small intestine. The disaccharide with an average molecular mass of 342 daltons is too large to pass through intact tight junctions. However, the disaccharide can penetrate disrupted tight junctions or tricellular contact points of epithelial cells, particularly if tricellulin is not sufficiently expressed to adequately seal the central channels of tricellular tight junctions (Krug et al. 2009). Additionally, it permeates epithelial apoptotic leaks and erosions. In case of intestinal barrier defects, the amount of disaccharide will rise relative to the amount of monosaccharide in the urinary samples taken after ingestion of the two sugars. The ratio of the two sugars, termed permeability index (PI), is a marker of intestinal integrity. An increased PI usually indicates impaired small intestinal permeability and disease. Mannitol is mostly taken as a test monosaccharide and lactulose as a test disaccharide.

After an overnight fast and the provision of a baseline morning urine sample, every participant drank a solution of 5 g lactulose, 10 g mannitol, and 20 g saccharose dissolved in 100 mL of water (Bjarnason et al. 1995). Urine was collected over a period of 5 h post dose in bottles containing sodium azide as a preservative. Participants continued fasting during the test period but were encouraged to drink water for 2 h post dose. HPLC analysis was done as described previously (Buhner et al. 2006; Haas et al. 2009).

Results are expressed as the percentage of urinary recovery of the ingested sugar. The permeability index (PI) is defined as the percentage recovery of lactulose divided by the percentage recovery of mannitol.

The sex distribution in the young age group and the old age group was markedly different. We considered this fact as negligible, because previous investigations (Kendall and Nutter 1970) and our own observation (unpublished) consistently showed that intestinal permeability parameters are not affected by gender.

### Blood measurements

Biochemical analysis of 81 older participants was done by a certified private medical laboratory in Berlin (Labor28, www.labor28.de). Blood vials were collected by the laboratory delivery service within 4 h after blood sampling and analyzed the same day.

The glomerular filtration rate was estimated with the logarithmic version of the Modification of Diet in Renal Disease Study Formula, MDRD formula (Levey et al. 1999) according to exp [5.228 – 1.154 × ln (serum creatinine mg/dL)−0.203 × ln (age in years)].

HsCRP values of 1.0 mg/L or more were defined as low‐grade inflammation based on the hsCRP threshold definitions established by the Centers of Disease Control and Prevention (CDC) and the American Heart Association (AHA; Pearson et al. 2003; Ballantyne and Nambi 2005).

### Statistics

Statistical analysis was carried out with SPSS version 19 (IBM Corp, Armonk, NY, USA), and a probability of error of 5% or less was considered statistically significant.

Descriptive results are given as means and standard deviations (SD) [range] if not indicated otherwise. The majority of results were not normally distributed; thus, consistent nonparametric tests were used for evaluation (Mann–Whitney *U* rank, Spearman rank coefficient). Linear regression analysis was done to assess the effect of age on the percentage of lactulose and mannitol excreted.

## Results

We evaluated 215 healthy people, who were divided into an older age group (*n *=**85, age 60–85 years) and a younger age group (*n *=**130, age 20–59 years). The participants had mean body mass indexes (BMI) representative for the respective age groups in Germany (Mensink et al. 2013; Table 1).

**Table 1.** Baseline characteristics and permeability results of 215 healthy volunteers

	All		*P*	Men		*P*
	Old	Young	O vs. Y	Old	Young	O vs. Y
Number	85	130		81	41	
Gender (% male)	96%	32%		100	100	
Age (years)	68 (4) [60–82]	34 (11) [18–59]	<0.001	69 (4) [60–82]	35 (10) [19–56]	<0.001
BMI (kg/m²)	26.5 (2.9) [20.0–33.5]	24.7 (3.5) [19.0–32.8]	<0.001	26.5 (2.9) [20.0–33.5]	24.7 (3.5) [19.6–32.7]	0.03
Perm Index ref.: ≤0.030	0.020 (0.011) [0.004–0.079]	0.019 (0.010) [0.007–0.081]	0.81	0.019 (0.011) [0.004–0.079]	0.018 (0.007) [0.007–0.038]	0.89
Lactulose (%) ref.: ≤0.044	0.26 (0.15) [0.04–0.92]	0.29 (0.13) [0.06–0.81]	0.02	0.25 (0.15) [0.04–0.92]	0.30 (0.15) [0.14–72]	0.03
Mannitol (%) ref.: ≤27.8	13.5 (4.1) [5.4–28.2]	16.4 (5.2) [3.6–28.8]	<0.001	13.5 (4.2) [5.4–28.2]	17.3 (5.5) [5.3–28.8]	<0.001
5 h urine volume (L)	0.68 (0.40) [0.15–2.30]	0.53 (0.34) [0.07–1.55]	0.003	0.68 (0.40) [0.15–2.30]	0.63 (0.43) [0.10–1.50]	0.28

BMI, body mass index; O, old; Y, young; ref., reference value; values are means (SD) [min–max].

As first step, we compared the permeability results between sexes in the younger age group (89 women/41 men), and found similar values for the permeability index [0.0188 (0.010) vs. 0.0182 (0.007), *P *=**0.668], the fractional urinary recovery of lactulose [0.279 (0.123) vs. 0.302 (0.145), *P *=**0.692], and the fractional urinary recovery of mannitol [14.9 (5.0) vs. 17.3 (5.5), *P *=**0.302]. To further exclude any sex‐related bias between the older age group and the younger age group, we decided to show all results also for men only (see e.g. Table 1).

Table 1 shows that the permeability index was similar in the older age group compared to the younger age group in all volunteers and in men only. However, the fractional urinary recovery of lactulose and of mannitol was significantly lower in the older age group (Table 1).

**Figure 1.**
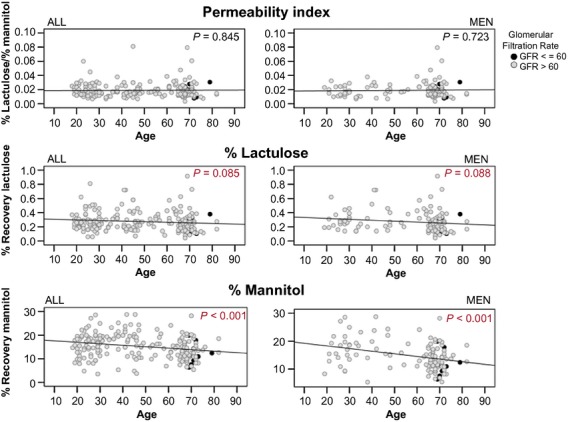
Permeability parameters with advancing age. The permeability index (= % lactulose/% mannitol) and the fractional urinary recovery of lactulose and mannitol are depicted with advancing age along with regression lines. Both lactulose and mannitol but not the permeability index trended to decrease with increasing age. Mildly impaired kidney function (glomerular filtration rate ≤ 60 mL/min) did not consistently lead to low recovery of test sugars as depicted by the black dots.

Regression analysis confirmed a stable permeability index with advancing age and the age‐dependent decline in lactulose or mannitol in the total group and in the group of men only (Fig. 1). The decline was similar to the results published by Saltzman et al. (1995) (Table 2). The decrease in lactulose trended consistently in the total sample, in men only, and in the historic results of Saltzman (all *P *=**0.09, see Table 2), whereas the decrease in mannitol was clearly significant in all three computations.

**Table 2.** Regression equations for permeability results. Table 2 shows regression equation for permeability results over age (18–82 years) for all participants and for men only as depicted in Fig. 1. The previously published historic results by Saltzman et al. (1995) were added for comparison. The number of women differed markedly in the three populations: all (43%), men only (0%) and Saltzman (80%). Despite the gender difference the development of permeability values along the age line was similar in all three computations speaking against any sexual dimorphism

	*N*	Regression equation	RC	*P*
Permeability index (% lact/% man)				
All	215	0.019 + 7*10^−6^*age	0.01	0.85
Men only	122	0.018 + 2*10^−5^*age	0.03	0.72
Saltzman et al. (1995)	56	–	–	–
Lactulose (%, urinary recovery)				
All	215	0.32−0.001*age	0.12	0.09
Men only	122	0.35−0.001*age	0.15	0.09
Saltzman et al. (1995)	56	0.21−0.001*age	0.23	0.09
Mannitol (%, urinary recovery)				
All	215	18.21−0.06*age	0.24	<0.001
Men only	122	20.18−0.09*age	0.33	<0.001
Saltzman et al. (1995)	56	14.00−0.06*age	0.27	0.05

RC, regression coefficient; % lact/% man, % urinary recovery of lactulose divided by % urinary recovery of mannitol.

**Figure 2.**
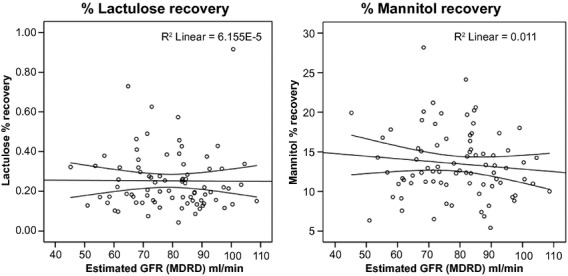
Bivariate correlation of sugar probes with glomerular filtration rate. No association was observed between the glomerular filtration rate and the fractional recovery of mannitol (*ρ *= −0.062, *P *=**0.627) or lactulose (*ρ *= −0.102, *P *=**0.426). The permeability index (*ρ *= −0.030, *P *=**0.818) did also not correlate with the glomerular filtration rate in the bivariate Spearman rank‐order correlation. GFR: glomerular filtration rate, MDRD. Formula according to the Modification of Diet in Renal Disease Study (Levey et al. 1999).

To evaluate possible reasons for the decreased excretion of test sugars with advancing age, we compared the urinary volume of younger and older people but could not identify the expected lower volumes in the older age group (Table 1, last line). In the younger age group, the urinary volume tended to be lower in women compared to men [0.480 (0.27) L vs. 0.624 (0.42) L, *P *=**0.057] resulting in a significant difference between the older and the younger age groups in the total population but not in men only. We further assessed kidney function in the older age group by means of the estimated glomerular filtration rate (GFR). GFR was not associated with the fractional recovery of lactulose or mannitol (Fig. 2), despite highly varying GFR values between 45 and 105 mL/min. However, this range is considered normal in the older age group [reference range 42–113 (Thomas 2008)]. We identified seven older participants with an estimated glomerular filtration rate lower than 60 mL/min. This mild renal impairment was not consistently associated with the lower excretion of lactulose or mannitol as depicted by the black dots in Fig. 1.

### Intestinal permeability and low‐grade inflammation in the older age group

Previous studies showed increased intestinal permeability with clinical inflammation (Soeters et al. 2007). We evaluated whether even subclinical low‐grade inflammation is associated with increased small intestinal permeability in older people and found similar results in participants with low‐grade inflammation and participants without low‐grade inflammation (Table 3).

**Table 3.** The older age group divided into participants with and without low‐grade inflammation

	Low‐grade inflammation		*P*‐value
	No	Yes	
Number of participants	35	46	
hsCRP (mg/L)	0.58 (0.22) [0.18–0.99]	3.37 (3.43) [1.01–15.7]	<0.001
Age (years)	69 (4) [65–82]	69 (4) [60–82]	0.84
Body mass index (kg/cm²)	25.5 (2.6) [20.8–31.2]	27.2 (2.9) [20.6–33.5]	0.009
Perm index	0.0170 (0.008) [0.0035–0.0477]	0.0211 (0.0133) [0.0076–0.0793]	0.16
Lactulose (% recovery)	0.224 (0.116) [0.043–0.626]	0.275 (0.166) [0.075–0.916]	0.19
Mannitol (% recovery)	13.4 (4.3) [6.9–28.2]	13.6 (4.3) [5.4–24.1]	0.77
Glomerular filtration rate (mL/min)	80 (13) [57–109]	78 (14) [45–103]	0.66
Lipid lowering medication *n* (%)	10 (28%)	14 (30%)	0.86
Antihypertensive medication *n* (%)	19 (54%)	28 (61%)	0.55
Type 2 diabetes *n* (%)	7 (20%)	11 (24%)	0.68

Low‐grade inflammation was defined as hsCRP ≥1 mg/L. Values are means (SD) [min–max].

**Figure 3.**
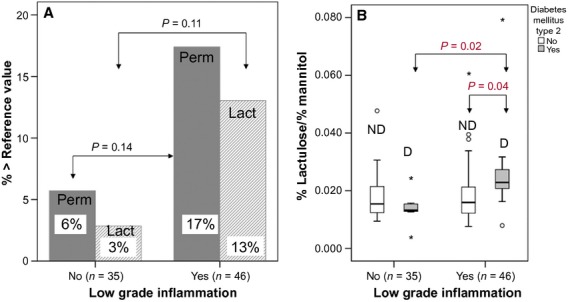
Small intestinal permeability in older people with and without low‐grade inflammation. Panel A: Proportion of participants above the reference range for permeability index (perm, >0.030) or lactulose recovery (lact > 0.44%) depending on the absence or presence of low‐grade inflammation. Panel B: ND: no diabetes, D = diabetes. Intestinal permeability is significantly increased in the copresence of type 2 diabetes with low‐grade inflammation (*n *=**11) compared to low‐grade inflammation (*n *=**35) or type 2 diabetes alone (*n *=**7), or in the absence of both (*n *=**28).

Nevertheless, the percentage of participants with increased permeability index was 10% higher in the group with low‐grade inflammation, which was statistically indifferent to the group with no inflammation (Fig. 3A). This increased permeability index was due to an increased lactulose recovery, fitting the pattern for a compromised intestinal barrier (Fig. 3A).

Medication and presence of type 2 diabetes did not differ between participants with and without low‐grade inflammation (Table 2). But the copresence of low‐grade inflammation coupled with type 2 diabetes resulted in significantly increased permeability values (0.027 ± 0.018) compared to participants with either low‐grade inflammation alone (0.019 ± 0.011, *P *=**0.043) or type 2 diabetes alone (0.014 ± 0.01, *P *=**0.015; Fig. 3B).

In total, 18.2% and 27.3% of the participants with diabetes and low‐grade inflammation had an increased permeability index or lactulose recovery, respectively, compared to none of the participants with diabetes only.

## Discussion

Our results clearly showed that the tightness of the epithelial small intestine barrier is not impaired by aging itself. This finding is of fundamental importance for drug and nutrition application in older people. Low‐grade inflammation did not significantly affect small intestine integrity on the population level, although it was associated with an about 10% higher prevalence of pathological permeability results compared to participants with no inflammation. However, low‐grade inflammation coupled with noninsulin dependent type 2 diabetes resulted in a significantly higher influx of lactulose and a clearly increased permeability index, which is typical for a compromised mucosal barrier.

### Small intestinal permeability does not change with advancing age

Three small previous investigations on the small intestinal permeability in older adults are available. In line with our results, all three studies concluded that the tightness of the small intestine barrier remains intact with advancing age (Saweirs et al. 1985; Beaumont et al. 1987; Saltzman et al. 1995). All three investigations were limited by one or more factors. Thus, further investigations into this topic were suggested by us and the authors of two recent review articles (Meier and Sturm 2009; Britton and McLaughlin 2013).

Saweirs et al. (1985) published their results on the l‐rhamnose and cellobiose test in 32 elderly inpatients compared to younger hospital volunteers in 1985. As Saweirs group used hospital patients, no firm conclusion can be drawn from their results for healthy older volunteers. Two years later in 1987, Beaumont and colleagues issued their study that included eight older healthy people undergoing the lactulose and mannitol test (Beaumont et al. 1987). In 1995, Saltzman et al. investigated another 17 older healthy people and compared the results to 39 younger adults also by means of the lactulose and mannitol test. Beaumont's and Saltzman's results are both limited by their small sample size. We were able to confirm the uncertain erstwhile and yet consistent evidence from previous studies in an adequately sized group of older people.

In line with our results, each of the three previous studies showed a decline in the absolute recovery rate of test sugars (Saweirs et al. 1985; Beaumont et al. 1987; Saltzman et al. 1995). Saltzman et al. (1995) reported the regression equation for this decline with data from US‐American citizens, which we almost exactly reproduced in our German population as detailed in the results section.

All previous studies attributed the decline in the absolute recovery of test sugars to the age‐related loss of renal function (Saweirs et al. 1985; Beaumont et al. 1987; Saltzman et al. 1995). The decline was correlated to creatinine clearance in one study (Saltzman et al. 1995) and the correction of urinary recoveries for creatinine clearance abolished group differences in another study (Beaumont et al. 1987). Yet, we did not observe any correlation with the estimated glomerular filtration rate, and the participants with the lowest glomerular filtration rate did not necessarily show low recovery of test sugars. This finding does not exclude the existence of such a relation. Kendall and Nutter (1970) showed that timed urinary excretion of intravenously administered xylose continuously decreases with advancing age, whereas intestinal absorption is maintained at a constant level. Xylose is an uncharged, nonmetabolized monosaccharide the size of mannitol. Thus, their excretion characteristics should be similar. But Kendall and Nutter (1970) also concluded that the correlation of sugar excretion with serum creatinine, creatinine clearance, or the estimated glomerular filtration rate is not close enough to use any of these values in an individual case. One reason that we could not show the correlation with renal function might be that glomerular filtration was still in the normal age range for all participants in our study.

Still, by focusing on the kidney function only, other factors might be overlooked. Several other premucosal and postmucosal factors exist that may affect the absolute amount of recovery of test sugars, particularly in older people, for example, later gastric emptying due to gastroparesis (Bjarnason et al. 1995). Even our active and overall healthy participants may have been affected by these conditions to some extent on the population level. We can exclude lower urine volume as a reason for lower recovery because mean urine volume tended to be even slightly higher in our older age group than in the younger age group. This finding was most likely caused by asking our older participants to drink sufficiently 2 h post dose to avoid inadequate urine production in this group. Even aging of the intestine itself might contribute to the decline, because it is still controversially discussed if the uptake of sugars is slightly lower in advanced age (Arora et al. 1989; Drozdowski and Thomson 2006).

### Intestinal permeability and low‐grade inflammation

Low‐grade inflammation is a risk factor for cardiovascular events (Kalogeropoulos et al. 2012) and is associated with obesity (Gentile et al. 2010), diabetes (Pradhan et al. 2001), metabolic syndrome, and a number of other chronic diseases.

Counteracting low‐grade inflammation may reduce the incidence of myocardial infarction or stroke (Kalogeropoulos et al. 2012), which would substantially contribute to cost containment within health systems. Low‐grade inflammation has also been related to aging per se (Chenillot et al. 2000; Cevenini et al. 2013), and more than 50% of older adults can be expected to have hsCRP concentrations of 1 mg/L and more (Imhof et al. 2003; Ahmadi‐Abhari et al. 2013). Thus, low‐grade inflammation is an important target for medical or nutritional therapies.

There is no general consensus on the threshold of hsCRP concentration to define low‐grade inflammation. We used the hsCRP concentration range of high sensitive C‐reactive protein published by the American Medical Association as moderate and severe risk of cardiovascular disease (Pearson et al. 2003). Clinically obvious inflammation is clearly associated with a disrupted intestinal barrier (Schulzke et al. 2009), but it was unclear whether minor inflammatory stimuli are associated with it. A 10% increase in the prevalence of increased permeability values has been observed with low‐grade inflammation in our study. This finding suggests that some older people are sensitive to minor inflammatory stimuli. When low‐grade inflammation is coupled with type 2 diabetes, the small intestine epithelial barrier worsened at the population level. This finding is the more interesting, as type 2 diabetes on its own is not associated with increased intestinal permeability (Secondulfo et al. 1999). It contrasts type 1 diabetes, where increased intestinal permeability is reported (Keita and Soderholm 2010).

## Limitations

The older age group consisted of predominantly males which may be seen as possible confounder of our results. It is generally accepted that intestinal permeability is similar in both sexes (Kendall and Nutter 1970) and this is in line with our laboratory observations over many years (unpublished). In the present report we reconfirmed the indifference in men and women in the younger age group. Furthermore, the permeability results in the total group were similar to the results in men only, which further strengthen the general significance of our findings.

The sample size in the subgroups of participants with diabetes or low‐grade inflammation is small and limits the significance of the results. Further research is thus necessary to confirm the effects of low‐grade inflammation coupled with chronic minor disease.

An increased prevalence of small intestinal bacterial overgrowth was previously reported in diabetic patients (Zietz et al. 2000; Rana et al. 2011) and this might have affected the permeability results. We can only speculate that bacterial overgrowth was most probably insignificant in our apparently healthy participants with well‐controlled and noninsulin‐dependent diabetes. If present, presumably, it should have led to increased bacterial degradation of lactulose already in the upper intestinal tract and thereby to a lower lactulose uptake and urinary excretion, which is contrary to our findings in diabetic participants.

We discussed measuring intestinal inflammation by fecal calprotectin concentration during the protocol development and decided against it because of two reasons. First, we considered calprotectin not sensitive enough to reflect a possible subclinical inflammation reflected by hsCRP concentration, which essentially is CRP in the reference range and slightly above (0.1–10 mg/L) analyzed with special methods to predict cardiovascular risk. Second, fecal calprotectin cannot differentiate between small intestinal and colonic inflammation, whereas lactulose/mannitol tests are indicative for small intestinal permeability only. Even if we experienced elevated calprotectin, we would not have been able to locate the intestinal inflammation to the small intestine. Nevertheless, a study published after the development of our study protocol reports fecal calprotectin being slightly elevated in healthy people aged 60 years and more (Joshi et al. 2010). Thus, it would be interesting in forthcoming studies to include fecal calprotectin also in healthy populations and correlate it with cardiovascular risk according to hsCRP.

A limitation might be that our results only represent small intestinal permeability. Lactulose and mannitol are degraded by the intestinal microbiota of the colon and yield no information on colonic permeability characteristics (Arrieta et al. 2006). Small intestinal permeability might differ from colon permeability. No exclusive in vivo marker of colonic permeability is available so far because both Cr‐EDTA and sucralose are stable throughout the GI tract and also provide information on the small intestine (Arrieta et al. 2006). The high variability in sucralose results and their missing association with small intestinal permeability or clinical outcome make it uncertain if this substance can provide any useful information beyond established permeability tests (Haas et al. 2009).

We also measured saccharose, the marker for gastric and duodenal permeability in all participants. Nevertheless, we decided against including these results in the present report, because gastric function in age can be affected by gastric atrophy or other factors, which we did not evaluate and which can limited the interpretation.

## Conclusion

We were able to confirm the results of previous studies and can now firmly conclude that the small intestinal barrier is not deteriorated in healthy aging. This finding is the fundamental clear message of this report and will be important for designing drug and nutrition strategies for older people. Based on our results, the reference values for intestinal permeability can be safely used in adults at least up to 80 years of age. The reason why the fractional recovery of test sugars gradually declines during aging is less clear. This question warrants further investigation, even if its pathophysiological relevance is still unclear.

Our results indirectly stress the importance of a healthy life style throughout life for maintaining an intact small intestinal mucosal barrier. Our data do not support the hypothesis that increased small intestinal permeability can contribute to low‐grade inflammation. But our results support the suggestion that low‐grade inflammation makes the intestinal barrier more vulnerable to insults from minor disease challenges.

## Acknowledgment

The current address of Verena Haas is: Charité – Universitätsmedizin Berlin, Experimental Clinical Research Center (ECRC), Charité ‐ Universitätsmedizin Berlin, Germany. The current address of Luzia Valentini is: University of Applied Sciences Neubrandenburg, Department of Dietetics, Broderaer Str 2‐4, 17033 Neubrandenburg. Monika Schoell is greatly acknowledged for her linguistic support.

## Conflict of Interest

None declared.
